# Twenty years of robotic surgery: a challenge for human limits

**DOI:** 10.1007/s13304-021-01071-x

**Published:** 2021-05-21

**Authors:** Ugo Boggi, Fabio Vistoli, Gabriella Amorese

**Affiliations:** 1 Division of General and Transplant Surgery, University of Pisa, Pisa, Italy; 2 Division of Anesthesia and Intensive Care, Azienda Ospedaliera Universitaria Pisana, Pisa, Italy

**Keywords:** da Vinci, Robotic surgery, Innovation, Development, Future perspectives

Leonardo Da Vinci means genius. Leonardo designed, and possibly built, the first articulated humanoid robot in 1495 in Milan (Italy) [1], but the term robot was coined only in 1921 by Karel Čapek, who staged a play with artificial people (“robota”) working for humans in a factory [2]. Indeed, robots embody one of the main human dreams: having machines replacing man in dangerous or heavy tasks. In keeping with this hope, robots are machines conceived to aid, augment, or substitute humans.

Starting from the last two decades of the twentieth century, laparoscopy allowed surgeons to perform many procedures formerly requiring large incisions (i.e., open surgery) through keyhole openings, thus proportionally reducing surgical trauma. In open surgery, relatively large incisions are required to achieve good exposure of the surgical site and confidently address diseased tissues. Laparoscopic surgery (LS) not only made this possible, but also improved clinical outcomes and became the standard technique for many surgical procedures. However, implementation of conventional LS was limited in some complex operations, such as pancreatoduodenectomy or resection of hilar cholangiocarcinoma because of bidimensional vision, loss of hand–eye alignment, use of rigid instruments with a fulcrum effect and only 4 degrees of freedom, and poor surgeon ergonomics [3]. Robotic surgery, as provided by the da Vinci Surgical System (dVSS) (Intuitive Surgical, Sunnyvale, CA, USA), has been the following revolution in surgery, because this system was specifically designed to address most of the technical limitations of conventional LS. The dVss is a master–slave telemanipulator that faithfully reproduces the movements of surgeon’s hands at tip of miniaturized intracorporeal instruments with seven degrees of freedom. Hand–eye coordination is also restored, thanks to an immersive view of the operative field that reproduces the natural alignment between vision, hands, and instruments. When all these features are taken together, the use of a dVSS restores the dexterity of open surgery in minimally invasive operations [4].

The dVSS was initially developed in the context of a military project of telesurgery, aiming to permit a remote surgeon to operate on wounded soldiers on a battlefield. The first robotic system was indeed integrated into a combat vehicle in 1994, and the first ex-vivo telesurgery procedure was performed during a combat exercise [5]. The first human operation, a cholecystectomy, was performed on March 3, 1997 by Himpens and Cadière [6]. In parallel with the dVSS, Zeus^®^, another robotic system, was developed by a competing company (Computer Motion^®^), but after several surgical procedures, this project was ended in 2003 when Computer Motion^®^ merged with Intuitive Surgical^®^. Several components of Zeus^®^ were integrated in the subsequent versions of the dVSS. The first dVSS was sold to the Leipzig Heart Center in Germany in late 1998. The device obtained FDA clearance in 2000 [2].

Table 1 reports several first-ever robotic abdominal procedures performed using the dVSS. Time distribution of these procedures demonstrates that in 2003, only 3 years after FDA clearance, there was a peak in the number of reported new robotic operations, demonstrating quick uptake and confident use of the new technology in several different areas. It is also worth to note that some of these procedures were truly complex, such as distal splenopancreatectomy, pancreatoduodenectomy, total gastrectomy with D2 lymphadenectomy, rectal anterior resection, transhiatal esophagectomy, right extended hepatectomy, and radical cystectomy with intra-abdominal formation of orthotopic ileal neobladder. Other procedures, such as robotic pancreas transplantation and selective distal splenorenal shunt for the treatment of severe portal hypertension, were so complex that were never performed using conventional LS. Geographical distribution of these first-ever procedures shows that nearly half of them were performed in the US and approximately one out of five in Italy (Fig. 1). As of December 31, 2019 5582 dVSS had been installed worldwide (3531 in the U.S., 977 in Europe, 780 in Asia, and 294 in the rest of the world). During 2019 over 1,200,000 dVSS surgeries were performed. Although in the common view, the dVSS is mostly used for urological procedures, according to US data, starting from 2018 general surgery procedures became prevalent [7]. General surgery is indeed the next great area of development of dVSS surgery. The need to use the robot in the wider anatomical field of general surgery, which increases variability and may include technically demanding procedures, has forced development of the system to increase flexibility in use.

**Table 1:** First world abdominal procedures performed using a da Vinci surgical system®

First author (ref)	Journal—volume—year	Location	Type of procedure
Himpens J	Surg Endosc—12—1998	Brussels, Belgium	Cholecystectomy
Cadière GB	Ann Chir—53—1999	Brussels, Belgium	Nissen fundoplication
Cadière GB	Obes Surg—9—1999	Brussels, Belgium	Gastric banding
Loulmet D	J Thorac Cardiovasc Surg—118—1999	Paris, France	Coronary artery bypass
Abou CC	J Urol—165—2001	Créteil, France	Radical prostatectomy
Guillonneau B	J Urol—166—2001	Paris, France	Nephrectomy
Weber PA	Dis Colon Rectum—45—2002	Hackensack, NJ, US	Sigmoid colectomy (benign disease)
Weber PA	Dis Colon Rectum—45—2002	Hackensack, NJ, US	Right hemicolectomy (benign disease)
Hashizume M	Surg Endosc—16—2002	Fukuoka, Japan	Ileocecal resection (cancer)
Hashizume M	Surg Endosc—16—2002	Fukuoka, Japan	Distal gastrectomy (cancer)
Hashizume M	Surg Endosc—16—2002	Fukuoka, Japan	Splenectomy
Hashizume M	Surg Endosc—16—2002	Fukuoka, Japan	Sigmoid colectomy (cancer)
Chapman WH 3rd	J Laparoendosc Adv Surg Tech A—12**—**2002	Greenville, NC, US	Splenectomy
Desai MM	Urology—60—2002	Cleaveland, OH, US	Adrenalectomy
Ballantyne GH	JSLS—7—2003	Hackensack, NJ, US	Ventral hernia repair
Menon M	BJU Int—92—2003	Detroit, MI, US	Nerve-sparing robot-assisted radical cystoprostatectomy
Melvin WS	J Laparoendosc Adv Surg Tech A.—13—2003	Columbus, OH, US	Distal splenopancreatectomy
Giulianotti PC	Arch Surg—138—2003	Grosseto, Italy	Pancreatoduodenectomy
Giulianotti PC	Arch Surg—138—2003	Grosseto, Italy	Liver segmentectomy
Giulianotti PC	Arch Surg—138—2003	Grosseto, Italy	Resection of esophageal leiomyoma
Giulianotti PC	Arch Surg—138—2003	Grosseto, Italy	Total gastrectomy with D2 lymphadenectomy
Giulianotti PC	Arch Surg—138—2003	Grosseto, Italy	Gastric wedge resection
Giulianotti PC	Arch Surg—138—2003	Grosseto, Italy	Resection of common bile duct
Giulianotti PC	Arch Surg—138—2003	Grosseto, Italy	Rectal anterior resection
Giulianotti PC	Arch Surg—138—2003	Grosseto, Italy	Partial splenectomy
Giulianotti PC	Arch Surg—138—2003	Grosseto, Italy	Repair of splenic artery aneurysm
Giulianotti PC	Arch Surg—138—2003	Grosseto, Italy	Renal artery aneurysmectomy and bypass
Melvin WS	Am J Surg—186—2003	Columbus, OH, US	Pancreaticojejenostomy (through an open access)
Horgan S	Am Surg—69—2003	Chicago, IL, US	Transhiatal esophagectomy
Molpus KL	JSLS-7—2003	Omaha, NE, US	Ovarian transposition
Vibert E	Arch Surg—138—2003	Paris France	Right extended hepatectomy
Beecken WD	Eur Urol—44—2003	Frankfurt am Main, Germany	Radical cystectomy with intra-abdominal formation of orthotopic ileal neobladder
Bentas W	World J Urol—21—2003	Frankfurt, Germany	Anderson-Hynes pyeloplasty
Kernstine KH	J Thorac Cardiovasc Surg—2004—2004	Iowa City, Iowa, US	2-stage, 3-field robotic esophagolymphadenectomy
Roeyen G	Surg Endosc—18—2004	Edegem, Belgium	Choledochotomy
Advincula AP	J Am Assoc Gynecol Laparosc—11—2004	Ann Arbor, MI, US	Uterine myomectomy
Killewich LA	Vasc Endovascular Surg.—38—2004	Galveston, TX, US	Aorto-femoral bypass for aortoiliac occlusive disease
Gettman MT	Urology—64—2004	Rochester, MI, US	Partial nephrectomy
Melamud O	Urology—65—2005	Orange, CA, US	Repair of vesicovaginal fistula
Mohr CJ	Arch Surg—140—2005	Stanford, CA, US	Roux-en-Y gastric bypass
Ryska M	Rozhl Chir—85—2006	Prague, Czech Republic	Robotic liver resection
Mufarrij PW	Rev Urol—8—2006	New York, NY, US	Ureterolysis for idiopathic retroperitoneal fibrosis
Yee DS	Urology—68—2006	Orange, California, US	Ureteroureterostomy
Sert B	Int J Med Robot—3—2007	Oslo, Norway	Radical hysterectomy
Jaik NP	J Gastrointestin Liver Dis—16—2007	Bethlehem, PA, US	Division of median arcuate ligament
Tayar C	Surg Endosc—21—2007	Créteil Cedex, France	Mesh repair of incisional hernia
Meehan JJ	J Pediatr Surg—42—2007	Iowa City, IA, US	Repair of congenital duodenal atresia
Korets R	Urology—70—2007	New York, NY, US	Ureterocalicostomy
Horgan S	Transplantation—84—2007	Chicago, IL, US	Segmental pancreas and kidney procurement for live donor pancreas–kidney transplantation
Meehan JJ	J Pediatr Surg—42—2007	Iowa City, IA, US	Repair of a Bochdalek congenital diaphragmatic hernia
Choi SB^a^	Yonsei Med J—49—2008	Seoul, South Korea	Left lateral sectionectomy
Vasile S^a^	Chirurgia (Bucur)—103—2008	Bucharest, Romania	Left lateral sectionectomy
Berry T	J Robot Surg—2—2008	Norfolk, VA, US	Vaginal construction in Mayer–Rokitansky–Küster–Hauser syndrome
Wahlgren CM	Ann Vasc Surg—22—2008	Chicago, IL, US	Repair of thoracoabdominal aortic aneurysm
Gundeti MS	Urology—72—2008	Chicago, IL, US	Augmentation ileocystoplasty and Mitrofanoff appendicovesicostomy
Liu C	J Minim Invasive Gynecol—15—2008	New York, NY, US	Partial bladder resection
Anderberg M	Eur J Pediatr Surg—19—2009	Lund, Sweden	Morgagni hernia repair
Martinez BD	Ann Vasc Surg—23—2009	Toledo, OH, US	Aorto-bifemoral graft bypass
Park JS	J Laparoendosc Adv Surg Tech A—19—2009	Seoul, South Korea	Resection of extra-adrenal pheochromocytoma
Kumar A	J Endourol—23—2009	New York, NY, US	Partial adrenalectomy
Vasilescu C	J Endourol—23—2009	Bucharest, Romania	Spleen-preserving distal pancreatectomy
Geffner SR	Reported online only—2009^b^	Livingston, NJ, US	Robotic kidney transplantation
Patriti A	J Hepatobiliary Pancreat Surg—16—2009	Spoleto, Italy	Simulatenous liver and colon resection
Bütter A	J Robot Surg—4—2010	London, Ontario, Canada	Duodenojejunostomy for superior mesenteric artery syndrome
Giulianotti PC	J Laparoendosc Adv Surg Tech A—20—2010	Chicago, IL, US	Extended hepatectomy plus hepaticojejunostomy for hilar cholangiocarcinoma
Giulianotti PC	Pancreas—40—2011	Chicago, IL, US	Total pancreatectomy
Zureikat	Arch Surg—146—2011	Pittsburgh, PA, US	Frey procedure
Buchs N	Int J Med Robot—7—2011	Chicago, Illinois, US	Palliation of unresectable pancreatic cancer
Boggi U	Transpl Int—26—2011	Palermo, Italy	Purely robotic live donor right hepatectomy
Giulianotti PC	Transpl Int—25—2012	Chicago, Illinois, US	Hand-assisted live donor right hepatectomy
Masrur M	JSLS—16—2012	Chicago, IL, US	Subtotal pancreas-preserving duodenectomy
Boggi U	Transplantation—93—2012	Pisa, Italy	Pancreas transplantation
Boggi U	Surgery—157—2015	Pisa, Italy	Distal selective spleno-renal shunt for severe portal hypertension

^a^These authors simultaneously reported the same procedure in August 2008

^b^https://www.itnnews.co.in/indian-transplant-newsletter/issue27/WORLDS-First-Robot-Assisted-Kidney-Transplant-Performed-656.htm

**Fig. 1:**
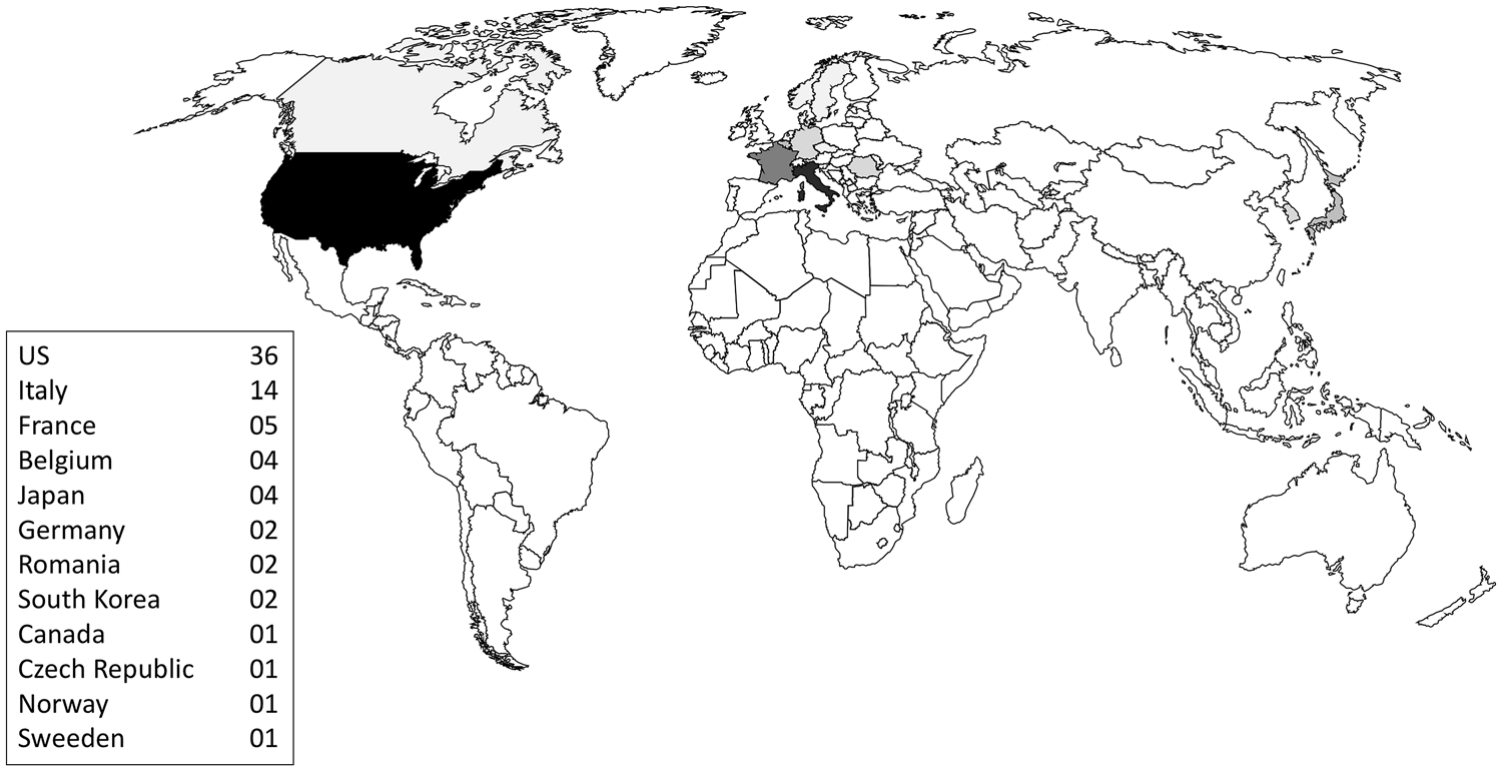
Number of first-ever reported robotic procedures by country. In the map, darker color represents higher number of first-ever reported robotic procedures in each country

The dVSS has still some technical limitations, mainly the need for a rigorous docking technique, longer operative times, lack of haptic feedback, and high costs. Indeed, robotic assistance clearly increases operative costs because of additional expenditures caused by device amortization and maintenance, acquisition of robotic instruments, and longer occupancy of operative room. Most of the robotic procedures are actually hybrid procedures requiring laparoscopic or thoracoscopic assistance, thus further increasing overall costs [8].

From a mechanical point of view, the dVSS is close to perfection and carries only a small risk of malfunction. A recent publication on 10,267 dVSS procedures reported a mechanical failure rate of 1.8% (185/10,267). Most of these malfunctions were caused by instrument failures (130; 70.3%) and were solved by replacing the malfunctioning instrument without consequences. In 7 patients, robotic malfunction required conversion to a different surgical approach (0.06%). Three patients were converted to laparoscopic surgery and four to open surgery. The overall mortality rate was 0.12% (12/10,267) [9].

By all the above mentioned features, it is clear that robotic assistance in surgery is essential, especially for complex procedures requiring fine intracorporeal dissections and multiple or delicate reconstructions. In competent and trained hands, the dVSS permits effortless performance of very difficult intracorporeal maneuvers and increases their reproducibility by different surgeons. Robotic assistance facilitates also training of newer generations of surgeons, thanks to the availability of the dual console and the immediate restoration of hand–eye coordination permitting also novices to faithfully reproduce surgical maneuvers under supervision. In addition, the advent of robotic surgery had also some indirect, although important and transversal, implications. First, the international community recognized that the optimal use of robotic technology requires the development of dedicated training pathways and that outcomes during the learning curve should be scrutinized [10]. Second, implementation of robotic surgery on a large scale for procedures believed to be safely feasible only through an open approach, promoted refinements in open surgical technique to keep up with minimally invasive standards (such as reduced blood loss, and precise anatomical dissections). Third, availability of robotic surgery has prompted improvements in key technology used in conventional LS (e.g., 4 K and 3D vision systems).

In conclusion, robots and robotic surgery are both here to stay. Current surgical residents, who start their training in operating rooms equipped with robots, will grow up using surgical robots such as millennials use smartphones. As it has already happened for laparoscopic cholecystectomy, future generations of surgeons might not be familiar in performing some procedures other than robotically. Operating in virtual reality, when eventually available, will create a new dimension of surgery permitting precise preoperative planning. Anticipation of operative scenarios could also allow assignment of tasks based on simulated performance. It is indeed clear that the concept of robotic surgery, that could also be renamed computer-enhanced surgery [3], carries the germ of additional disruptive innovations that are expected to expand surgeon power beyond human capability. Additional and fascinating scenarios include integration of multiple technologies in a single surgical instrument, navigation, artificial intelligence, and autonomous robotic function.

As many patents hold by Intuitive Surgical will expire shortly, and other companies are developing newer devices, the market is expected to become competitive eventually reducing costs of robotic assistance. This will certify the final rise of robots in surgery. The intuition of Leonardo over 500 years ago is going to be turned into reality.

## Data Availability

All materials are available upon request.
